# Legume NADPH Oxidases Have Crucial Roles at Different Stages of Nodulation

**DOI:** 10.3390/ijms17050680

**Published:** 2016-05-18

**Authors:** Jesús Montiel, Manoj-Kumar Arthikala, Luis Cárdenas, Carmen Quinto

**Affiliations:** 1 Institute of Biochemistry, Biological Research Center of the Hungarian Academy of Sciences, 6726 Szeged, Hungary; jesuyuz@gmail.com; 2 Escuela Nacional de Estudios Superiores, Universidad Nacional Autónoma de México (UNAM), León, Blvd. UNAM 2011, León 37684, Guanajuato, Mexico; manojarthik@gmail.com; 3 Departamento de Biología Molecular de Plantas, Instituto de Biotecnología, Universidad Nacional Autónoma de México, UNAM, Apartado Postal 510-3, Cuernavaca 62271, Morelos, Mexico; luisc@ibt.unam.mx

**Keywords:** legumes, RBOH, reactive oxygen species (ROS), rhizobia, symbiosis

## Abstract

Plant NADPH oxidases, formerly known as respiratory burst oxidase homologues (RBOHs), are plasma membrane enzymes dedicated to reactive oxygen species (ROS) production. These oxidases are implicated in a wide variety of processes, ranging from tissue and organ growth and development to signaling pathways in response to abiotic and biotic stimuli. Research on the roles of RBOHs in the plant’s response to biotic stresses has mainly focused on plant-pathogen interactions; nonetheless, recent findings have shown that these oxidases are also involved in the legume-rhizobia symbiosis. The legume-rhizobia symbiosis leads to the formation of the root nodule, where rhizobia reduce atmospheric nitrogen to ammonia. A complex signaling and developmental pathway in the legume root hair and root facilitate rhizobial entrance and nodule organogenesis, respectively. Interestingly, several reports demonstrate that RBOH-mediated ROS production displays versatile roles at different stages of nodulation. The evidence collected to date indicates that ROS act as signaling molecules that regulate rhizobial invasion and also function in nodule senescence. This review summarizes discoveries that support the key and versatile roles of various RBOH members in the legume-rhizobia symbiosis.

## 1. Introduction

NADPH oxidases (NOX) are flavin-containing enzymes specialized in the production of reactive oxygen species (ROS). These membrane-localized proteins catalyze the reduction of oxygen to generate superoxide anions using the reducing power of NADPH. NOX enzymes, also known as respiratory burst oxidases, were initially identified in human phagocytes, which mediate the oxidative burst in response to microbes or inflammatory stimuli. NOX members have been identified and studied in species ranging from fungi to mammals. In plants, the genes encoding these enzymes display tissue-specific expression profiles and have particular functions in key processes, such as growth and development (of underground and aerial tissues/organs), the innate response, and signaling in response to abiotic stress responses [1,2].

The plant NADPH oxidases, referred to as respiratory burst oxidase homologs (RBOHs), are approximately 100 kD in size and possess six transmembrane regions. The third and fifth transmembrane domains coordinate a heme group through four histidine residues. Flavin- and NADPH-binding motifs are located at the carboxy (C)-terminus, while the amino (N)-terminus contains regulatory sequences and two EF-hand motifs [3,4]. RBOH-mediated ROS production is stimulated when calcium ions interact with the EF-hand motifs. In addition, the activity of these enzymes is positively or negatively modulated at the N-terminus by protein kinases, calcium-dependent protein kinases, nitric oxide, and complexes formed between calcineurin B-like calcium sensors 1/9 and protein kinases [5]. RBOH gene families generally consist of a large and variable number of members [6]. Historically, functional analyses of RBOHs in plant-microbe interactions have focused mainly on defense mechanisms after pathogen attack and plant tissue damage; nonetheless, recent discoveries have unraveled their crucial participation in modulating the legume-rhizobia symbiosis [7,8,9]. The beneficial association between legumes and the Gram-negative soil bacteria, rhizobia, leads to the formation of novel organs, the root nodules, where the intracellular bacteria develop into nitrogen-fixing bacteroids. This mutualistic relationship begins in the rhizosphere with species-specific molecular crosstalk between both symbionts that involves (iso)-flavonoids released by the legume root and lipo-chitooligosaccharides, known as nodulation factors (NFs), produced and secreted by rhizobia in the rhizosphere [10]. When compatible NFs are perceived by receptors in root hairs, a specific signaling pathway is triggered that leads to cellular, physiological, and morphological changes that promote root hair infection by the rhizobia via an infection thread (IT). Simultaneously, cortical cell division and further nodule development take place in the root cortex. During this process, rhizobia are released from the IT into the nodule cells and the bacteria become bacteroids that catalyze the conversion of nitrogen to ammonia [11]. Several groups have demonstrated that RBOHs are essential during nodulation [7,8,9]. This review describes the participation of RBOHs in the legume-rhizobia symbiosis, highlighting their contribution to the symbiotic signaling pathway, rhizobial infection, nodule organogenesis, and senescence.

## 2. RBOHs Function Downstream of Nodulation Factor (NF) Perception

**Figure 1:**
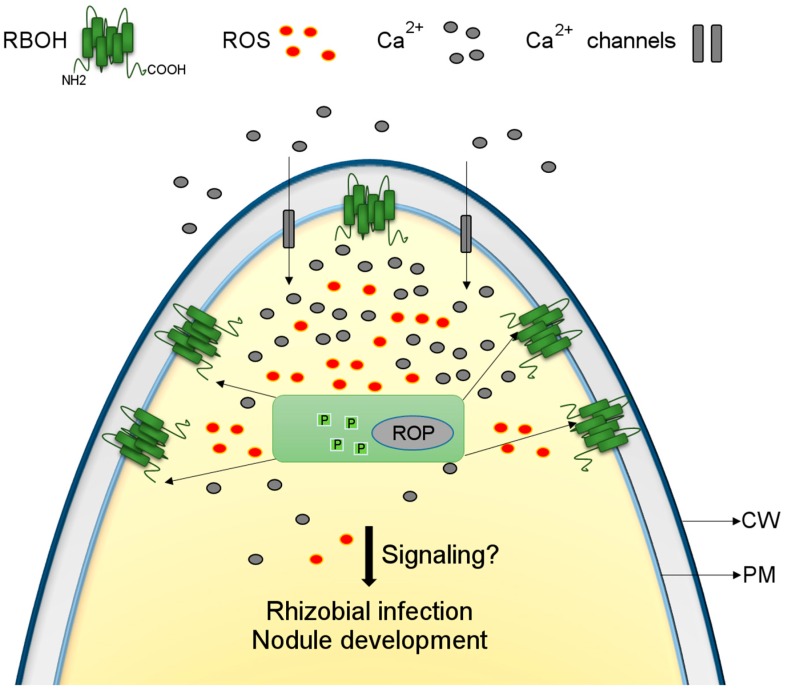
Scheme of the early symbiotic signaling pathway after nodulation factor (NF) recognition by root hairs. NF induces a transient increase in respiratory burst oxidase homologues (RBOH)-dependent reactive oxygen species (ROS) production and cytoplasmic calcium concentration in the apical region of legume root hairs. RBOH activity is presumably regulated by Rho-like GTPases from plants (ROP GTPases) and phosphorylation at its N-terminus. ROS produced by RBOH activation likely act as signaling molecules that mediate rhizobial infection and nodule development. CW, cell wall; PM, plasma membrane.

During NF perception by the legume root hairs, physiological and molecular reprogramming occurs that is necessary for rhizobial infection and nodule organogenesis [12]. This reprogramming results in changes in the root hair tip zone, such as increased levels of calcium ions, ROS production, ion fluxes, cell-membrane depolarization, cytoplasm alkalinization, perinuclear calcium oscillations, cytoskeleton rearrangements, and gene expression changes [13,14,15,16,17,18,19,20,21]. The transient ROS burst in NF-treated *Phaseolus vulgaris* root hairs, which occurs within seconds of NF application and is maintained for approximately 3 min, is one of the fastest responses recorded in this signaling pathway [19]. The finding that this response is sensitive to a NADPH oxidase inhibitor (dyphenilene iodonium, DPI) suggests that RBOH participates in the legume-rhizobia symbiosis. The fast and transient oxidative burst contrasts with the sustained increase in ROS levels when legume root hairs are treated with a pathogen elicitor, which culminates in root hair death. Furthermore, no changes in ROS levels were detected when the root hairs were challenged with a similar concentration of non-active NFs (*N*-acetyl glucosamine pentamers), indicating that compatible NFs trigger specific RBOH-dependent ROS changes in the root hairs. This notion is supported by findings reported by Morieri *et al.* [22] involving *nodL*-Nod Factors in *Medicago truncatula* root hairs. The absence of a NodL-determined acetyl group impaired calcium influx, but did not affect calcium spiking. These authors proposed that two pathways are triggered in the root hair after activation of NF receptors by compatible NFs, with the first pathway resulting in calcium spiking and the second stimulating RBOH activity and increased calcium influx. The latter pathway is most likely responsible for rhizobial infection [22]. Although the mechanism underlying RBOH activation during this process is unknown, it has been shown that conformational changes at the N-terminus of RBOH induced by calcium binding stimulate ROS production [23,24,25,26,27]. The increase in cytoplasmic calcium levels in the apical region of root hairs correlates spatially and temporally with the transient oxidative burst [17,19]. Therefore, feedback is likely to occur at this early stage of the signaling process, since ROS produced by RBOHs can promote the opening of calcium channels (Figure 1) [28]. However, further modulation is required to control ROS homeostasis, since sustained levels would lead to cell death of the root hair. This assumption is supported by findings in *Pisum sativum* plants. Glyan’ko and Ischenko [29] detected an increase in NADPH oxidase activity (3.9-fold with respect to non-inoculated roots) in microsomal fractions of roots from *P. sativum* seedlings at 5 min post-inoculation with *R. leguminosarum*. In addition, they found that this response is prevented by amiodarone and lanthanum chloride treatment, which activates and blocks calcium channels, respectively. These data indicate that NADPH oxidase activation requires calcium channel opening, but that excessive calcium has a negative effect on NADPH oxidase activity. Recently, nitric oxide (NO) has been proposed as another negative regulator of RBOH activity in the symbiotic process [30]. This assumption is based on the evidence of NO production at different time-points in nodulation and its capacity to abolish AtRBOHD activity during the hypersensitive response in *A. thaliana* leaves [31]. Thus, RBOH is only transiently activated after NF treatment and rhizobial inoculation.

*Rbohs* are part of a large gene family in legumes, and their transcripts are differentially expressed in organs and tissues and at various developmental stages [8,9,32,33,34,35]. However, RBOHs involved in the early steps of the NF-signaling program remain to be identified. *RbohA* and *RbohF* have been proposed as potential candidates, since they are the most abundant *Rboh* transcripts in *P. vulgaris* and *M. truncatula* root hairs, respectively [8,36]. However, in *P. vulgaris*, *RbohB* seems to have a central role, since rhizobial infection is impaired in transgenic plants in which *PvRbohB* is silenced by RNAi and the protein is located in the apical zone of growing root hairs in WT plants [9].

Several studies place RBOHs as positive regulators that function early in the signaling pathway after NF treatment in legume root hairs [11,19,22], and other reports indicate that these oxidases must be switched off at a subsequent stage. Specific NFs induce root hair tip swelling in legumes at 1 h post-incubation. This morphological response correlates with a decrease in ROS levels together with the down-regulation of *MtRboh2* and *MtRboh3* (*MtRbohE* and *MtRbohB*, respectively, [8]) transcripts in *M. truncatula* roots [37,38]. The hypothesis that *Mt*RBOHs are deactivated during root hair tip swelling is supported by pharmacological evidence, since a similar morphological effect is observed in *M. truncatula* root hairs at 1 h post-incubation with DPI [37]. The decrease in ROS production following NF treatment was observed in *M. truncatula* mutants affected in calcium spiking (*dmi1*, *dmi2*, *dmi3*, *nsp1*, *nsp2*, and *hcl*) but not in *nfp*, which lack calcium influx [38]. Nonetheless, after NF-induced swelling, root hairs resume polar growth and become branched at 12 h post-incubation. RBOHs seem to be involved in this process, since branching of *M. truncatula* root hairs is impaired in plants in which the Rho-like GTPases from plants (ROP GTPase), MtROP9, a putative activator of RBOHs, is silenced [39].

## 3. RBOHs Mediate Infection Thread (IT) Progression and Nodule Organogenesis

Legume root hair cells are the gateway for rhizobial invasion; however, the infection process is preceded by the arrest of root hair growth and its subsequent curling to entrap the microsymbiont within an infection pocket. Thereafter, the IT is formed by an invagination process of the cell wall and the plasma membrane in the root hair [40]. The IT is internalized to reach the nodule primordia, via polarized growth that resembles root hair development, but in an inward direction. Nitroblue tetrazolium blue (NBT) staining revealed the presence of superoxide anions in the tips of growing root hairs of *Arabidopsis thaliana* plants [41]. Likewise, superoxide anions accumulate to high concentrations in the infection pocket and IT of *Medicago sativa* and *M. truncatula* root hairs inoculated with *Sinorhizobium meliloti* [42,43]. In *P. vulgaris*, *Pv*RBOHB is thought to underlie superoxide accumulation, since a GFP-RBOHB chimera (driven by the CaMV 35S promoter) was detected by confocal microscopy in the infection pocket and IT of *P. vulgaris* root hairs infected with rhizobia (Supplementary Video S1). Pharmacological approaches provide initial evidence of the involvement of RBOH at this nodulation stage, since root hair curling and rhizobial invasion of root hairs are prevented by previous incubation of *M. sativa* roots with DPI [44]. Nonetheless, RBOH participation was conclusively demonstrated in *P. vulgaris* transgenic roots expressing an RNAi-*PvRbohB* construct. Loss-of-function of *RbohB* resulted in aborted ITs at the base of the root hairs in *P. vulgaris* plants inoculated with *Rhizobium tropici*. However, downregulation (~60%) of the *PvRbohB* transcript did not impact the number of infection events per root [9]. Surprisingly, a five-fold over-expression of *PvRbohB* induced an increase in the number of ITs per root, but did not affect IT progression [7]. The lack of IT progression can be explained by the fact that post-transcriptional mechanisms control RBOHB function during the formation and migration of the IT within *P. vulgaris* root hair cells. In *A. thaliana*, targeting of RBOHC to the root hair tip is crucial for its role in root hair development and depends on vesicle trafficking, ROP GTPases, and actin microfilaments [23]. A similar scenario exists in the legume-rhizobia symbiosis, since rhizobial infection is affected in *M. truncatula* plants silenced in ROP9. This Rac1 GTPase is presumably a positive regulator of RBOH function in *M. truncatula* [39]. Furthermore, mutations in genes involved in actin rearrangements trigger abortion of the IT within root hairs [45,46,47]. The lines of evidence generated to date show strong similarities between root hair development and IT progression; therefore, it is reasonable to assume that legumes recruit molecular components implicated in root hair growth during IT progression. For instance, filamentous actin plus ends accumulate both at the tip of root hairs during apical growth and in the infection pocket of *P. vulgaris* root hairs inoculated with rhizobia [48]. Similarly, the secretory vesicles that usually are deposited in root hair tips are relocated to the tips of growing ITs [49]. These vesicles are expected to support IT progression together with other molecules that are necessary for root hair development, such as calcium, ROS, and the actin cytoskeleton. In that regard, RBOH-mediated ROS production likely participates in cell wall remodeling during IT advancement. ROS can have opposite effects on cell wall extensibility. Hydrogen peroxide promotes cell wall rigidity by polymerizing phenolic compounds in a cell wall peroxidase-dependent reaction, whereas hydroxyl radicals promote cell wall loosening by cleaving cell wall polymers [50]. Such processes are crucial for IT initiation and migration, and this notion is further supported by the observation that IT progression is arrested in a pectate lyase (*Lj*NPL) mutant of *Lotus japonicus* inoculated with *Mesorhizobium loti* [51]. Taken together, these data indicate that RBOH-mediated ROS production is required in root hair-rhizobial infections but also during IT progression.

**Figure 2:**
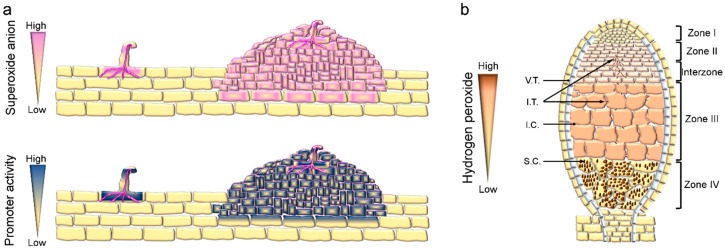
ROS production during nodule development and functioning. (**a**) Superoxide accumulation in the IT and nodule primordium of *P. vulgaris* (pink staining) correlates with the promoter activity of *PvRbohB* (blue staining) [9,52]. Superoxide is also generated in nodule primordia from indeterminate nodules [42]; (**b**) hydrogen peroxide is detected *in vivo* from Zone II to Zone III in *M. truncatula* nodules with the ROS-sensor Hyper [43]. Similarly, hydrogen peroxide is produced in the IT and senescent cells (cerium chloride detection method [54,55]). I.C., infected cell; I.T., infection thread; V.T., vascular tissue; S.C., senescent cell. Zone I, meristematic region; Zone II, infection zone; Zone III, nitrogen-fixing cells; Zone IV, senescent region.

IT progression is accompanied by another key event in the nodulation process, namely nodule primordium formation. Development of the nodule primordium requires reactivation of mitotic activity in the root cortex cells [9]. NBT staining revealed that superoxide anions accumulated to high concentrations in the nodule primordia of *M. sativa* plants infected with *S. meliloti* [42]. Similar results were found in the nodule primordia of *P. vulgaris* plants inoculated with *R. tropici* using the same approach [52]. *Pv*RBOHB is most likely the enzyme responsible for increased levels of superoxide anions in *P. vulgaris*, since the spatial and temporal distribution of this anion correlate with the histochemical staining pattern of *P. vulgaris* plants expressing the promoter-GUS transcriptional fusion of *PvRbohB* [9] (Figure 2A). Moreover, transgenic *P. vulgaris* plants with *PvRbohB* loss-of-function exhibited cortical cell divisions after rhizobial inoculation; however, because the number of nodules in *PvRbohB*-RNAi plants is drastically reduced, ROS are probably needed for nodule development [9]. The superoxide generated by RBOH seems to have a prominent role in the mitosis required for nodule primordium formation, although its precise function during nodule primordium development is unclear. ROS play versatile roles in the biomechanics of plant cell walls and changes in cell wall biomechanics are essential for nodule formation. For instance, the superoxide anion in *A. thaliana* roots is linked to cell proliferation in the meristematic region, while hydrogen peroxide seems to have a role in the elongation zone, where cell differentiation occurs [53]. The balance between ROS production and accumulation is central for root growth and, similarly, nodule organogenesis is a ROS-dependent process.

## 4. RBOHs Impact Nodule Function and Senescence

**Figure 3:**
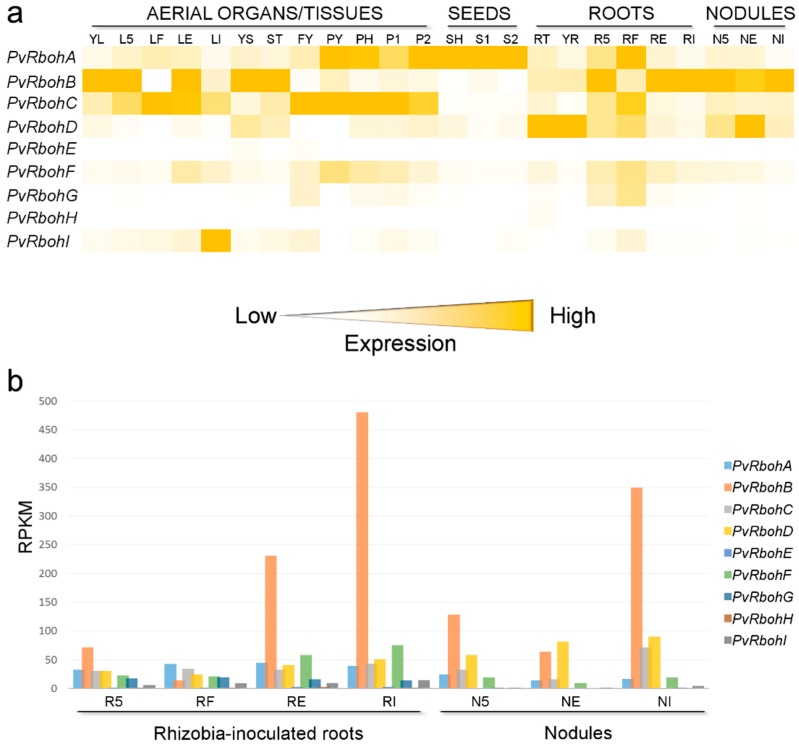
Expression profile of *Rboh* genes in different organs and tissues of *P. vulgaris*. (**a**) Heat map expression profiles highlight the most abundant *Rboh* transcripts in different organs and tissues of *P. vulgaris*; (**b**) *PvRboh* expression under different conditions in rhizobia-inoculated roots and nodules; *PvRbohB* is predominantly expressed; nonetheless, other *Rboh* transcripts are expressed in nodules such as *PvRbohA*, *PvRbohC*, and *PvRbohD*. The expression analysis was assessed in the *Phaseolus vulgaris* Gene Expression Atlas [35,59]. YL—Fully expanded second trifoliate leaf tissue from plants provided with fertilizer; L5—Leaf tissue collected five days after plants were inoculated with effective rhizobium; LF—Leaf tissue from fertilized plants collected at the same time as LE and LI; LE—Leaf tissue collected 21 days after plants were inoculated with effective rhizobium; LI—Leaf tissue collected 21 days after plants were inoculated with ineffective rhizobium; YS—All stem internodes above the cotyledon collected at the second trifoliate stage; ST—Shoot tip, including the apical meristem, collected at the second trifoliate stage; FY—Young flowers, collected prior to floral emergence; PY—Young pods, collected 1 to 4 days after floral senescence. Samples contain developing embryos at the globular stage; PH—Pods approximately 9 cm long, associated with seeds at heart stage (pod only); P1—Pods between 10 and 11 cm long, associated with stage 1 seeds (pod only); P2—Pods between 12 and 13 cm long, associated with stage two seeds (pod only); SH—Heart stage seeds, between 3 and 4 mm across and approximately 7 mg; S1—Stage 1 seeds, between 6 and 7 mm across and approximately 50 mg; S2—Stage 2 seeds, between 8 and 10 mm across and between 140 and 150 mg; RT—Root tips, 0.5 cm of tissue, collected from fertilized plants at the second trifoliate stage of development.; YR—Whole roots, including root tips, collected at the second trifoliate stage of development; R5—Whole roots separated from five-day old pre-fixing nodules; RF—Whole roots from fertilized plants collected at the same time as RE and RI; RE—Whole roots separated from fix+ nodules collected 21 days after inoculation; RI—Whole roots separated from fix- nodules collected 21 days after inoculation; N5—Pre-fixing (effective) nodules collected five days after inoculation; NE—Effectively fixing nodules collected 21 days after inoculation; NI—Ineffectively fixing nodules collected 21 days after inoculation.

**Figure 4:**
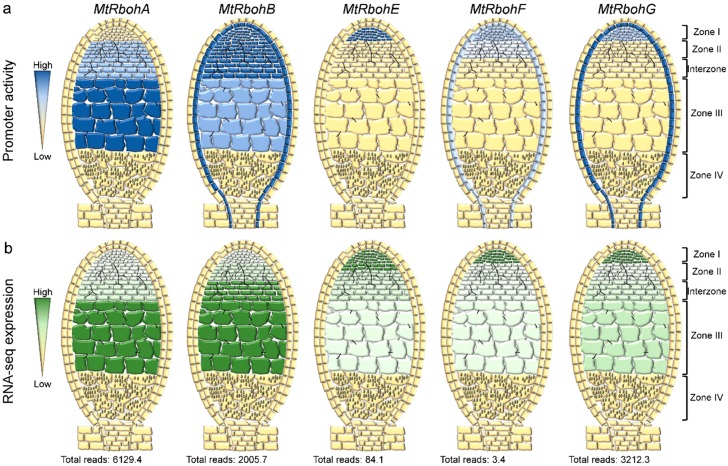
Promoter activity and gene expression of *Rbohs* in different zones of *M. truncatula* nodules. A, scheme of the promoter activity (blue staining [8]) (**a**) and RNA-Seq expression profile (green) [34] (**b**) of *MtRboh* genes in different regions of the nodules suggests the involvement of several *MtRbohs* in nodule functioning. RNA-Seq analysis was not performed in the senescent cells. Zone I, meristematic region; Zone II, infection zone; Zone III, nitrogen-fixing cells; Zone IV, senescent region.

Successful rhizobial invasion and nodule organogenesis culminate in the intracellular colonization of the nodule cells by the endosymbionts liberated from branched ITs. When bacteria enter root nodule cells, they become surrounded by a plant-derived membrane, known as the peri-bacteroidal membrane, which encloses the intracellular bacteria in a symbiosome. Within the symbiosome, the rhizobia differentiate into bacteroids that are able to fix N_2_. The nitrogenase complex expressed by the bacteroids catalyzes the reduction of dinitrogen into ammonia under hypoxic conditions, since the enzyme is irreversibly inactivated by oxygen [11]. Surprisingly, a recent report demonstrated that hydrogen peroxide is produced in the nitrogen-fixing zone of *M. truncatula* nodules expressing the ROS-sensor Hyper [43] (Figure 2b). Previously, histochemical staining with cerium chloride revealed that hydrogen peroxide accumulates in the matrix and cell wall of ITs in mature nodules of *M. sativa* and *P. sativum* [42,54] (Figure 2b). In addition, DPI incubation prevents deposition of cerium perhydroxides in the ITs, suggesting a key role for RBOHs in this process [54]. When nitrogen fixation peaks (21 days post-inoculation, dpi) several *Rboh* transcripts start to accumulate, with *MtRbohA* and *PvRbohB* genes exhibiting the highest transcript accumulation in *M. truncatula* and *P. vulgaris* nodules, respectively [8,9,33,34,35] (Figure 3). In addition, an analysis of the promoter activity and RNA-sequencing (RNA-Seq) of laser-dissected tissue revealed a high transcriptional activity of *MtRbohA* in the nitrogen fixation zone of *M. truncatula* nodules [8,34] (Figure 4). Approaches based on RNAi gene silencing and gain-of-function of *Rboh* genes confirmed their role in nodule functioning in *M. truncatula* and *P. vulgaris* plants [7,8,9]. Down-regulation (>60%) of *MtRbohA* transcript levels does not affect nodule formation in *M. truncatula* plants, but does result in a 25% reduction in nitrogen fixation. Such a decrease seems to be related to the low accumulation of the transcript levels of the bacterial genes *nifD* and *nifH*, which encode the Mo-Fe and Fe proteins of the nitrogenase complex, respectively [8]. Similarly, acetylene reduction is remarkably impaired in the small and few nodules of *P. vulgaris* in which *PvRbohB* is silenced by RNAi. Moreover, the ITs within the nodules are thicker and the symbiosome integrity is altered upon *PvRbohB* silencing [9]. By contrast, over-expression of *PvRbohB* causes a substantial increase in nitrogen fixation at 21 (>2-fold) and 30 (>3-fold) dpi. The increased acetylene reduction is certainly a consequence of other alterations found within the nodules, such as the high number of bacteroids per symbiosome and poly-β-hydroxybutyrate granules per bacteroid [7]. The evidence collected so far indicates that RBOH has a pivotal role in nodule functioning that correlates with the distribution patterns of ROS within the nodule. The phenotypes observed suggest that the ROS produced by RBOHs act as signaling molecules to the endosymbiont and also mediate the remodeling of the cell wall in the IT. However, other *Rboh* members probably have different functions in nodule organogenesis, since *PvRbohA*, *PvRbohC*, and *PvRbohD* transcripts are also abundant in *P. vulgaris* nodules [9] (Figure 3). Likewise, *MtRbohB* shows strong promoter activity in the meristematic region, infection zone, and nitrogen-fixing zone of *M. truncatula* nodules. By contrast, *MtRbohE* and *MtRbohF* are mainly expressed in the meristematic region, and *MtRbohG* in the vascular tissue of nodules [8] (Figure 4). RNA-Seq of different zones of *M. truncatula* nodules further confirmed the particular expression pattern of each *Rboh* and their putative participation in different zones of the nodule [34] (Figure 4).

Proper nodule development and functioning rely on sophisticated regulation of the redox state; this requires the participation of antioxidant molecules and RBOH-mediated ROS production [56]. A shift in redox homeostasis seems to be responsible for senescence during nodule aging, as this process is characterized by a decrease in the content of antioxidant compounds and an increase in oxidized biomolecules, which negatively impact nitrogen fixation [57]. At the ultrastructural level, the senescent nodules of *Glycine max* and *P. sativum* plants exhibit alterations in symbiosome structure, accompanied by the presence of hydrogen peroxide, supporting the notion that ROS have important roles also during nodule senescence [54,55,58] (Figure 2b). Furthermore, the drastic reduction in nitrogen fixation in *P. vulgaris* nodules silenced in *PvRbohB* is probably an early senescence phenotype of the nodules, since the symbiosome structure is clearly affected [9]. By contrast, over-expression of *PvRbohB* (*PvRbohB*-OE) delays senescence of the nodule. Interestingly, in *PvRbohB*-OE nodules, acetylene reduction levels remain elevated at 30 dpi, while in the control plants, nitrogen fixation decreases during nodule senescence [7].

Studies based both on silencing and over-expression of *PvRbohB* indicate that this oxidase has a central role during nodule senescence, and this notion is further supported by the strong promoter activity of this gene in senescent nodules [7]. The collected evidence suggests that ROS-produced by RBOH has a signaling role during nodule senescence in *P. vulgaris*, acting as a negative regulator. Conversely, none of the *MtRbohs* (*MtRbohA*, *MtRbohB*, *MtRbohE*, *MtRbohF*, and *MtRbohG*) analyzed to date show promoter activity in the senescence zone of indeterminate *M. truncatula* nodules, eliminating the possibility that these genes function in nodule senescence in this legume [8] (Figure 4). It seems likely that RBOHs have a more prominent role in determinate nodules than in indeterminate nodules. However, the possibility that *MtRboh* genes participate in nodule senescence should not be discarded, since uncharacterized members of the family could be involved in this process.

## 5. Conclusions and Perspectives

A diverse range of approaches undertaken by different laboratories have demonstrated that RBOH members participate at distinct stages of the legume-rhizobia symbiosis. These oxidases play key roles throughout nodulation; for instance, they are involved in the signaling pathway induced by NF treatment in the root hairs, IT growth, nodule organogenesis, nitrogen fixation, and nodule senescence. The evidence collected to date indicates that ROS generated by RBOH activity function as signaling molecules in the nodulation pathway, but also modify the structure of the cell wall in the IT. However, important gaps remain in our understanding of the function of RBOHs at several stages of rhizobial symbiosis. Specifically, the localization and dynamics of NADPH oxidases throughout nodulation remain to be determined. In addition, only two members of the *Rboh* gene family, *MtRbohA*, and *PvRbohB*, have been functionally analyzed, although transcriptome evidence suggests the involvement of other *Rbohs* both in *M. truncatula* and *P. vulgaris* nodules. Therefore, functional characterization of other *Rboh* members is urgently needed. Another remaining challenge is to decipher the molecular targets of the ROS produced in response to RBOH activity, as well as the precise function of RBOHs in the NF signaling cascade. The legume-rhizobia symbiosis is a remarkable model for understanding ROS participation at the molecular level during infectious and developmental processes, and information gleaned from studies conducted in this system will be of general interest to the plant-microbe interaction research community.

## Supplementary Materials

Supplementary materials can be found at http://www.mdpi.com/1422-0067/17/5/680/s1, Video S1: Subcellular localization of PvRBOHB-GFP in the infection thread of *P. vulgaris* roots.

## Abbreviations

NOX	NADPH oxidases
ROS	Reactive oxygen species
RBOHs	Respiratory burst oxidase homologs
NFs	Nodulation factors
IT	Infection thread
NBT	Nitroblue tetrazolium blue
